# Genome-wide analysis of maize *OSCA* family members and their involvement in drought stress

**DOI:** 10.7717/peerj.6765

**Published:** 2019-04-11

**Authors:** Shuangcheng Ding, Xin Feng, Hewei Du, Hongwei Wang

**Affiliations:** 1 Agricultural College, Yangtze University, Jingzhou, China; 2 Hubei Collaborative Innovation Center for Grain Industry, Yangtze University, Jingzhou, China; 3 College of Life Science, Yangtze University, Jingzhou, China

**Keywords:** Phylogenetic analysis, OSCA gene family, Genetic variation, Drought stress, Maize, Expression pattern

## Abstract

**Background:**

Worldwide cultivation of maize is often impacted negatively by drought stress. Hyperosmolality-gated calcium-permeable channels (OSCA) have been characterized as osmosensors in *Arabidopsis*. However, the involvement of members of the maize *OSCA* (*ZmOSCA*) gene family in response to drought stress is unknown. It is furthermore unclear which *ZmOSCA* gene plays a major role in genetic improvement of drought tolerance in Maize.

**Methods:**

We predicted the protein domain structure and transmembrane regions by using the NCBI Conserved Domain Database database and TMHMM server separately. The phylogeny tree was built by Mega7. We used the mixed linear model in TASSEL to perform the family-based association analysis.

**Results:**

In this report, 12 *ZmOSCA* genes were uncovered in the maize genome by a genome-wide survey and analyzed systematically to reveal their synteny and phylogenetic relationship with the genomes of rice, maize, and sorghum. These analyses indicated a relatively conserved evolutionary history of the *ZmOSCA* gene family. Protein domain and transmembrane analysis indicated that most of the 12 ZmOSCAs shared similar structures with their homologs. The result of differential expression analysis under drought at various stages, as well as the expression profiles in 15 tissues, revealed a functional divergence of *ZmOSCA* genes. Notably, the expression level of *ZmOSCA4.1* being up-regulated in both seedlings and adult leaves. Notably, the association analysis between genetic variations in these genes and drought tolerance was detected. Significant associations between genetic variation in *ZmOSCA4.1* and drought tolerance were found at the seedling stage. Our report provides a detailed analysis of the *ZmOSCAs* in the maize genome. These findings will contribute to future studies on the functional characterization of ZmOSCA proteins in response to water deficit stress, as well as understanding the mechanism of genetic variation in drought tolerance in maize.

## Introduction

Drought stress is a key limiting environmental factor which can eventually cause food and societal problems, and therefore numerous studies have been devoted to unraveling the mechanisms of drought resistance in plants. Studies over the past decades have found that plant responses to drought stress mainly include the perception, regulation, and transmission of signals through various pathways, as well as the regulation of stress-responsive gene expression (Ingram & Bartels, 1996; Zhu, 2002; Bartels & Sunkar, 2005). As a consequence, physiological and morphological modifications result to address the stress. During the signal perception, especially during osmotic changes, calcium concentrations have been found to be elevated (Knight, Trewavas & Knight, 1997; McAinsh & Pittman, 2009). Importantly, the increase in the concentration of Ca^2+^ occurs within 5 s, which might be the earliest detectable event in plants under stress (Knight, Trewavas & Knight, 1997). During the last three decades, studies have shown that Ca^2+^ is an important second messenger in the signal transduction process when plants respond to biotic and abiotic stress (Reddy, 2001; Hepler, 2005; DeFalco, Bender & Snedden, 2009). Ca^2+^-permeable channels have been proposed to function as osmosensors in bacteria and animals stimulated by osmotic/mechanical stress (Booth et al., 2007; Arnadottir & Chalfie, 2010). Therefore, it can be inferred that Ca^2+^ is crucial for both sensing external osmotic stress and activating many signal transduction pathways.

Studies were carried out to find genes regulating early response to stress, especially the concentrations of calcium. When investigating the gene expression profile in *Arabidopsis* subjected to drought conditions, 16 early responses to dehydration (*ERD*) genes were reported to be identified after 1 h of drought stress (Kiyosue, Yamaguchi-Shinozaki & Shinozaki, 1994). Amongst the 16 *ERDs*, *ERD4*, harboring a highly conserved DUF221 domain (Pfam accession: 02714), was conserved between species (Camargo et al., 2007; Liu et al., 2009; Rai et al., 2012). Recently, using a gene screening strategy based on calcium imaging, an *Arabidopsis* hyperosmolality-gated calcium-permeable channel (OSCA) mutant was identified; *OSCA1* showed a low hyperosmolality-induced Ca^2+^ increase (OICI), and *OSCA1* was further characterized to be an osmosensor in *Arabidopsis* (Yuan et al., 2014). Interestingly, *OSCA1* also contains the DUF221 domain. The results disclose that DUF221 may participate in osmotic adjustment.

Association analysis, based on linkage disequilibrium (LD) and excavation of genetic variation, have been utilized as a novel strategy for dissecting the genetic basis of complex traits in crops (Yu & Buckler, 2006). Drought tolerance is a complex and intrinsic trait, and the identification of favorable alleles for drought tolerance in maize through association studies, including candidate gene association analysis, is an ever-growing research area. Due to the rapid decrease in LD, association analysis in the maize genome could screen genetic variations at a single gene level. Although several genetic variations were found to enhance drought resistance (Liu et al., 2013; Mao et al., 2015; Wang et al., 2016; Xiang et al., 2017), allelic variations underlying drought tolerance still need to be identified to facilitate molecular breeding.

It has been shown that *OSCA1* acts as an osmosensor in *Arabidopsis* (Yuan et al., 2014), and the role of the *OSCA* gene family is essential for plants to respond to stress. Recently, genome-wide analysis of genes containing the DUF221 domain were performed in rice to understand its possible roles (Li et al., 2015; Ganie, Pani & Mondal, 2017). Therefore, in this study, we characterized the *OSCA* family members in the maize genome and analyzed the phylogenetic and syntenic relationship amongst these *OSCA*s. In addition, we studied the expression profiles of *OSCA*s under drought stress and performed a family-based genome-wide association study. These results could be applied for further functional research of *ZmOSCA*s and raise our understanding of the roles of plant *OSCA*s in drought stress.

## Materials and Methods

### Identification of OSCA protein-coding genes in the maize genome

Conserved OSCA domain DUF221 (Pfam accession: 02714) from the Pfam database (Finn et al., 2014) was used to build the Hidden Markov Model-based searches (http://hmmer.janelia.org/) and scanned against the maize genome (genome assembly: AGPv3) and the sorghum genome (genome assembly: V3). All the retrieved sequences were curated using the NCBI Conserved Domain Database (www.ncbi.nlm.nih.gov/Structure/cdd/wrpsb.cgi) (Marchlerbauer et al., 2011) to determine whether the proteins harbor the DUF221 domain or not.

### Genic structure and phylogenetic relationships analysis

The protein sequences of the identified maize and sorghum OSCAs were downloaded from Phytozome v10.0. The protein sequence of previously identified OSCA protein-coding genes in the Arabidopsis and rice genome (Yuan et al., 2014; Li et al., 2015) were also downloaded form Phytozome v10.0. To show the exon/intron structure, the coding sequence of each OSCA gene were aligned to its corresponding genomic sequence and then a schematic representation was generated using GSDS 2.0 (http://gsds.cbi.pku.edu.cn) (Hu et al., 2015). To construct a phylogenetic tree of the identified OSCA proteins in rice, *Arabidopsis*, and sorghum, and maize genome, ClustalW (Thompson, Higgins & Gibson, 1994) was used to align multiple protein sequences of OSCA. The phylogenetic tree was constructed using this alignment output based on a neighbor-joining method in MEGA7 (Kumar, Stecher & Tamura, 2016) using the following parameters: Poisson correction, pairwise deletion, uniform rates, and bootstrap (1,000 replicates).

### Prediction of transmembrane region

TMHMM Server V2 was used to predict the transmembrane region (TMs) of ZmOSCAs, and the prediction of TMs was manually edited according to the TMs of OSCA1.1, OSCA1.2, and OsOSCA1.2 (Jojoa-Cruz et al., 2018; Liu, Wang & Sun, 2018; Maity et al., 2018; Murthy et al., 2018; Zhang et al., 2018).

### Gene duplication analysis

Chromosomal locations of *ZmOSCAs* were collected from the maize genome (assemble version: AGPv3) in Phytozome v10.0. The duplicated segmental blocks and gene pairs were analyzed on the Plant Genome Duplication Database (available online: http://chibba.agtec.uga.edu) with a display distance of 100 kilobases.

### Expression profile analysis

Seedlings growth conditions and drought treatments of B73 (maize inbred line) were conducted according to Wang et al. (2016). Hydraulically cultured three-leaf stage seedlings were put on a plate and subjected to dehydration (40–60% relative humidity and 28 °C). As for the effect of drought implied on adult leaf, drought stress was applied by withholding water after the eight-leaf stage (V8), with well-watered plants (soil water content 40%) as control. The middle section of flag leaf which came from three replicates, were collected at the 12-leaf stage (V12), the 14-leaf stage (V14), the 16-leaf stage (V16), and the silking stage (R1) for both drought stressed and well-watered plants as control. Leaf samples from at least three replicates were frozen by liquid nitrogen and then stored at −80 °C before RNA isolation.

Raw RNA was extracted from leaf samples using TRI Reagent (Invitrogen, Carlsbad, CA, USA) according to the product manuals. The relative expression of *ZmOSCAs* was quantified using quantitative real time-PCR (qRT-PCR). qRT-PCR was tested in 96-well plates using an ABI7500 Real-Time PCR Systems (Applied Biosystems, Foster City, CA, USA). The PCR reaction system consists of one μl cDNA, 200 nM primers, and five μl SYBR Premix Ex Taq II (Takara, Beijing, China), and the reaction volume was 10 μl. The PCR reaction was performed with the following conditions: 10 min at 94 °C, 40 cycles of 15 s at 94 °C, and 30 s at 60 °C. The internal control was the expression of *ZmUbi-2* (UniProtKB/TrEMBL; ACC: Q42415). The quantification method used was 2^−ΔCT^, and the variation in the expression was derived from three biological replicates.

### Association analysis

Association analysis for *ZmOSCAs* was conducted by using a mapping population containing 367 maize inbred lines and corresponding drought resistance phenotypes reported previously (Wang et al., 2016). The mapping population contains 556k single nucleotide polymorphism (SNP) markers, and the minor allele frequency of each marker was greater than or equal to 0.05. All identified *ZmOSCAs* harbored 168 SNPs in the coding region and both the 5′-, and 3′-untranslated region. Three statistical models including the general linear model (GLM) model, adjusting the first two principal components (PC_2_), and the mixed linear model (MLM) model (incorporating PC_2_ and a Kinship matrix) were selected to identify the SNPs significantly associated with drought resistance by using the TASSEL4.0 program (Yu et al., 2006; Bradbury et al., 2007).

## Results

### Identification of the *OSCA* family members in maize

We carried out a systematic genome-wide screen of putative *OSCA* genes in maize. Initially, a Hidden Markov Model search was performed against the maize genome (genome version: AGPv3.0) utilizing the DUF221 domain (Pfam accession: 02714). Ultimately, 12 genes were found to be *ZmOSCA*s in the maize genome and named according to the *Arabidopsis* orthologs (Table 1). The physical location of each *ZmOSCA* in the genome was identified according to physical coordination provided by MaizeGDB (https://www.maizegdb.org). A total of 12 *ZmOSCA* genes were distributed unevenly on all the 10 chromosomes except chromosomes 2, 4, 7, and 10, without any clustering. Chromosomes 1 and 3 possessed as many as three *ZmOSCA* genes (the largest number of *OSCA* genes on a chromosome), and chromosomes 5 and 8 equally contained two genes. In contrast, chromosomes 6 and 9 each only harbored one *ZmOSCA*. The exon number of *ZmOSCA*s varied from 1 to 11. Approximately 75% (8/12) of the *ZmOSCA* genes contained more than eight exons, and only 17% (2/12) of the genes had less than two exons.

**Table 1:** Detailed information for twelve *ZmOSCA* genes in the *Zea mays* L. genome.

Gene name	Gene identifier	Chromosome	Protein length (aa)	ORF (bp)	Number of exons	Class
*ZmOSCA1.1a*	GRMZM2G064189	3	327	984	4	1
*ZmOSCA1.1b*	GRMZM2G021194	3	768	2,307	11	1
*ZmOSCA1.2*	GRMZM2G456000	8	768	2,307	11	1
*ZmOSCA1.3*	GRMZM2G181206	6	748	2,247	11	1
*ZmOSCA1.4*	GRMZM2G128641	1	810	2,433	11	1
*ZmOSCA2.1*	GRMZM2G163059	3	586	1,761	8	2
*ZmOSCA2.2*	GRMZM2G409093	1	765	2,298	10	2
*ZmOSCA2.3*	GRMZM2G164470	5	749	2,250	10	2
*ZmOSCA2.4*	GRMZM2G039186	1	699	2,100	10	2
*ZmOSCA2.5*	GRMZM2G402708	8	706	2,121	10	2
*ZmOSCA3.1*	GRMZM2G162253	5	249	750	2	3
*ZmOSCA4.1*	GRMZM2G059891	9	796	2,391	1	4

### Phylogenetic relationship analysis of *OSCA* genes

**Figure 1:**
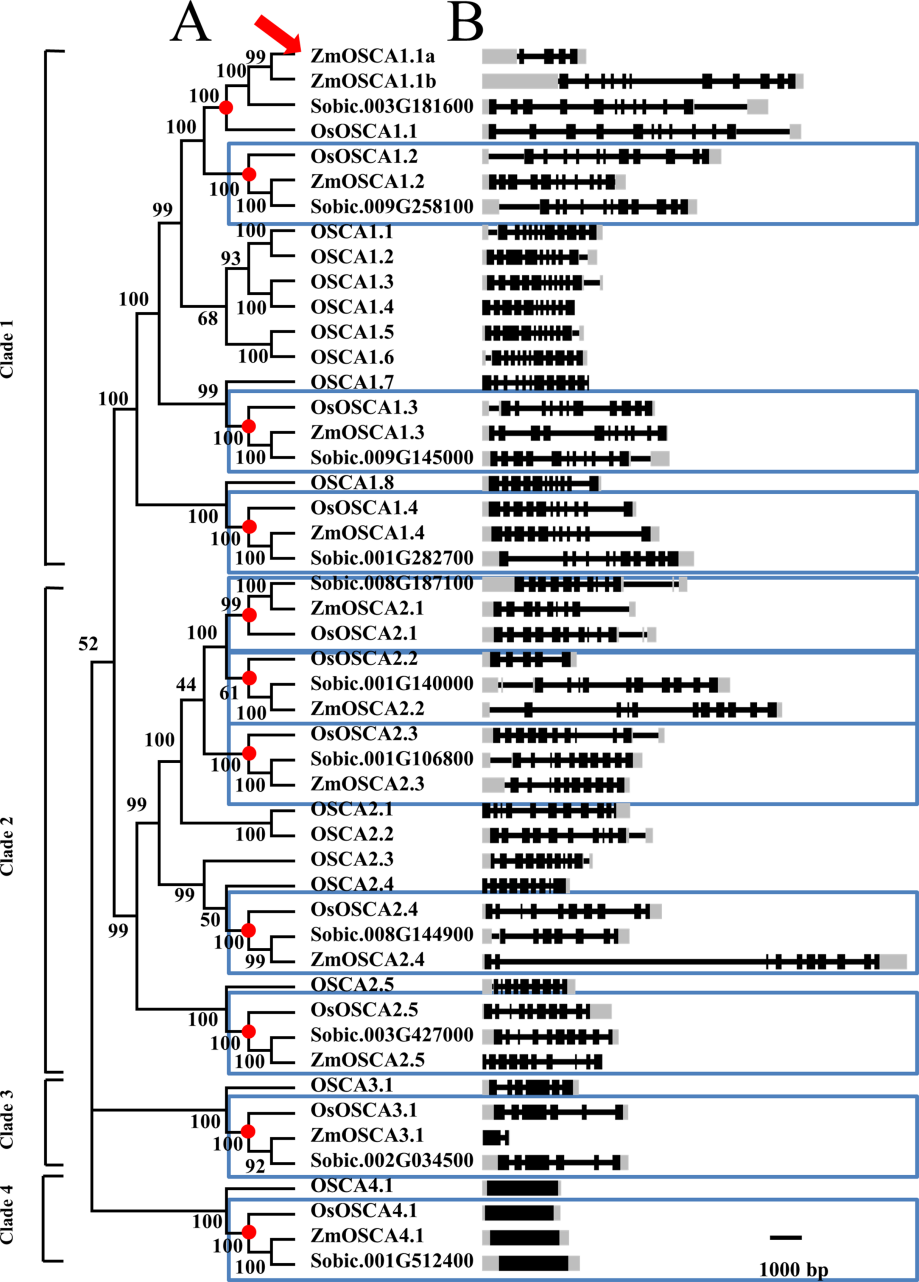
Phylogenetic tree and gene structure of predicted OSCA genes from maize, rice, sorghum, and Arabidopsis. (A) The phylogenetic tree of 49 full-length OSCA protein sequences from four species. The names used for *OSCA* gene in rice and Arabidopsis are according to Li et al. (2015). Each node was labeled with bootstrap values from 1,000 replicates. Genes contained in a blue-box were regarded as direct orthologous genes among species. (B) Position of exons, introns, and (untranslated region) UTR in the OSCA genes. Introns are indicated by lines, exons by black boxes, UTR by gray box.

In order to elucidate the phylogenetic relationships among *OSCA*s, a neighbor-joining tree of ZmOSCA proteins and the corresponding orthologs from rice, sorghum, and *Arabidopsis* was built, and the tree was based on the alignment of full-length OSCA proteins (Fig. 1; Table S1). As shown on the phylogenetic tree, 49 *OSCA* genes can be classified into four main classes: clades 1, 2, 3, and 4 (Fig. 1). All *Arabidopsis* and rice *OSCA*s fell in the same class or clade as previously reported, which is in agreement with previous work (Yuan et al., 2014). Interestingly, proteins derived from dicotyledonous *Arabidopsis* clustered separately from those of three monocot plants. Also, we found that some proteins from rice, sorghum, and maize displayed pairwise correspondence (blue box in Fig. 1), not only indicating that these genes are phylogenetically conserved among these species, but demonstrating that maize and sorghum possessed a closer phylogenetic relationship compared to maize and rice, conforming to the perspective that sorghum is a closer relative to maize than rice.

Throughout the phylogenetic tree, clades 1, 2, 3, and 4 contain 21, 20, 4, and 4 OSCA proteins, respectively (Fig. 1). Amongst the 49 *OSCAs*, there were 5, 5, 1, and 1 *ZmOSCAs* in clades 1, 2, 3, and 4, respectively. However, for the rice and sorghum genomes, there were 4, 5, 1, and 1 *OSCAs* in clades 1, 2, 3, and 4, respectively (Table 2). Obviously, compared with rice and sorghum, maize possessed one more *OSCA* in clade 1 (Fig. 1). Meanwhile, we detected the nodes that lead to maize-, sorghum-, and rice-specific clades (red circles in Fig. 1). These nodes denote the divergence point between maize, sorghum, and rice, and therefore reveal the most recent common ancestral (MRCA) genes before the split. After MRCA analysis, there were 11 clades (indicated by red circles in Fig. 1), and nearly all the clades were constituted by one gene from the rice, maize, and sorghum genomes. However, there was just one clade that contained two genes from the maize genome (indicated by the red arrow in Fig. 1). The results showed that between the maize, sorghum, and rice genomes, the *OSCA* gene family shared a similar evolutionary history, whilst the maize genome possessed a duplication event leading to a gain in one gene (indicated by red arrow in Fig. 1) after the maize, sorghum, and rice genomes split.

**Table 2:** Numbers and classification of *OSCA* genes in *Arabidopsis*, rice, maize, and sorghum.

Species	Clade 1	Clade 2	Clade 3	Clade 4	In total
*Arabidopsis thaliana*	8	5	1	1	15
*Oryza sativa*	4	5	1	1	11
*Zea mays*	5	5	1	1	12
*Sorghum bicolor*	4	5	1	1	11

### Synteny of *OSCA*s in maize, sorghum, and rice genomes

**Figure 2:**
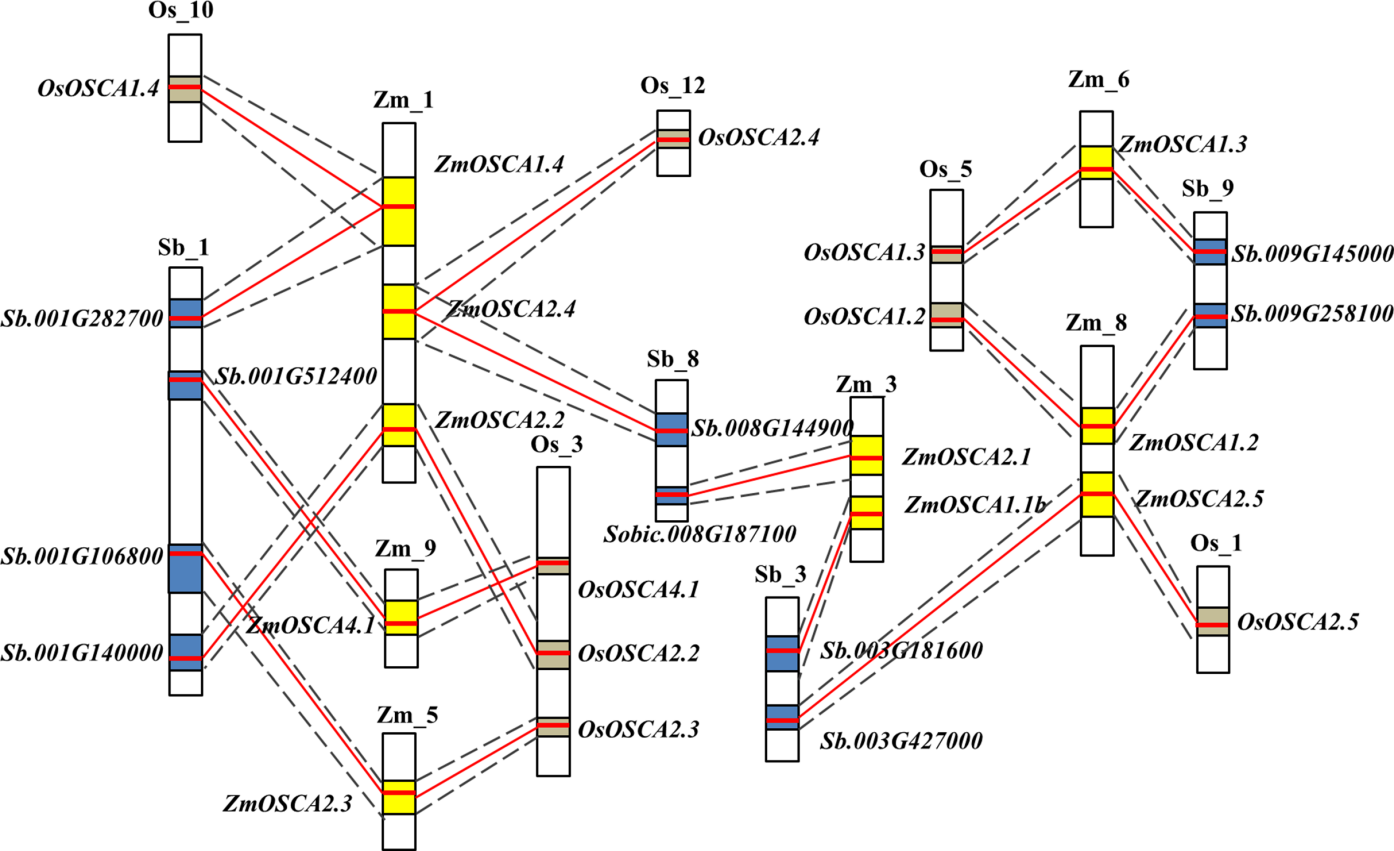
Schematic diagram of syntenic chromosomal segments containing *ZmOSCA* genes between the sorghum, rice, and maize genomes. *Zm*, *Os*, and *Sb* stand for chromosomes in maize, rice, and sorghum, respectively. The homologous chromosomal regions of different genomes are connected by black dotted lines Each OSCA homologous gene pair is linked by a red line. Yellow, blue, and brown boxes represent the homologous regions within the maize, rice, and sorghum genome.

Gene collinearity comparative genomics indicates homologous gene function and phylogenetic relationships amongst several species and even the genome organization of extinct ancestral species. Thus, we investigated the colinearity of *OSCA* genes in rice, sorghum, and maize genomes. Initially, gene colinearity data were retrieved from the Plant Genome Duplication Database using *ZmOSCA*s as anchors, then the chromosomal segment containing multiple homologous genes across species was defined as a genomic syntenic block. After this analysis, 10 *ZmOSCAs* were found to have collinearity, and the syntenic members or collinear genes in rice and sorghum were shown in Fig. 2. As a result, eight chromosomal segments containing *OSCA*s were identified as being evolutionally conserved between rice, maize, and sorghum; these include *ZmOSCA1.2*, *ZmOSCA1.3*, *ZmOSCA1.4*, *ZmOSCA2.2*, *ZmOSCA2.3*, *ZmOSCA2.4*, *ZmOSCA2.5*, and *ZmOSCA4.1* (Fig. 2; Table S2). In addition, genes within the eight syntenic block amongst the maize, sorghum, and rice genomes were identical to the phylogenetic analysis (Fig. 1), confirming the accuracy of our analysis. Syntenic blocks of *ZmOSCA1.1b* and *ZmOSCA2*.1b were observed between maize and sorghum genomes respectively, but no syntenic blocks of these genes were found between maize and rice genomes. Meanwhile, no collinear segments of *ZmOSCA1*.1a and *ZmOSCA3*.1 were found in the genome of maize, rice, and sorghum. The results suggest that most of the *ZmOSCA*s existed before the divergence of species, but some *ZmOSCA*s may have originated from duplication of the maize genome.

### The conserved domain of ZmOSCAs

**Figure 3:**
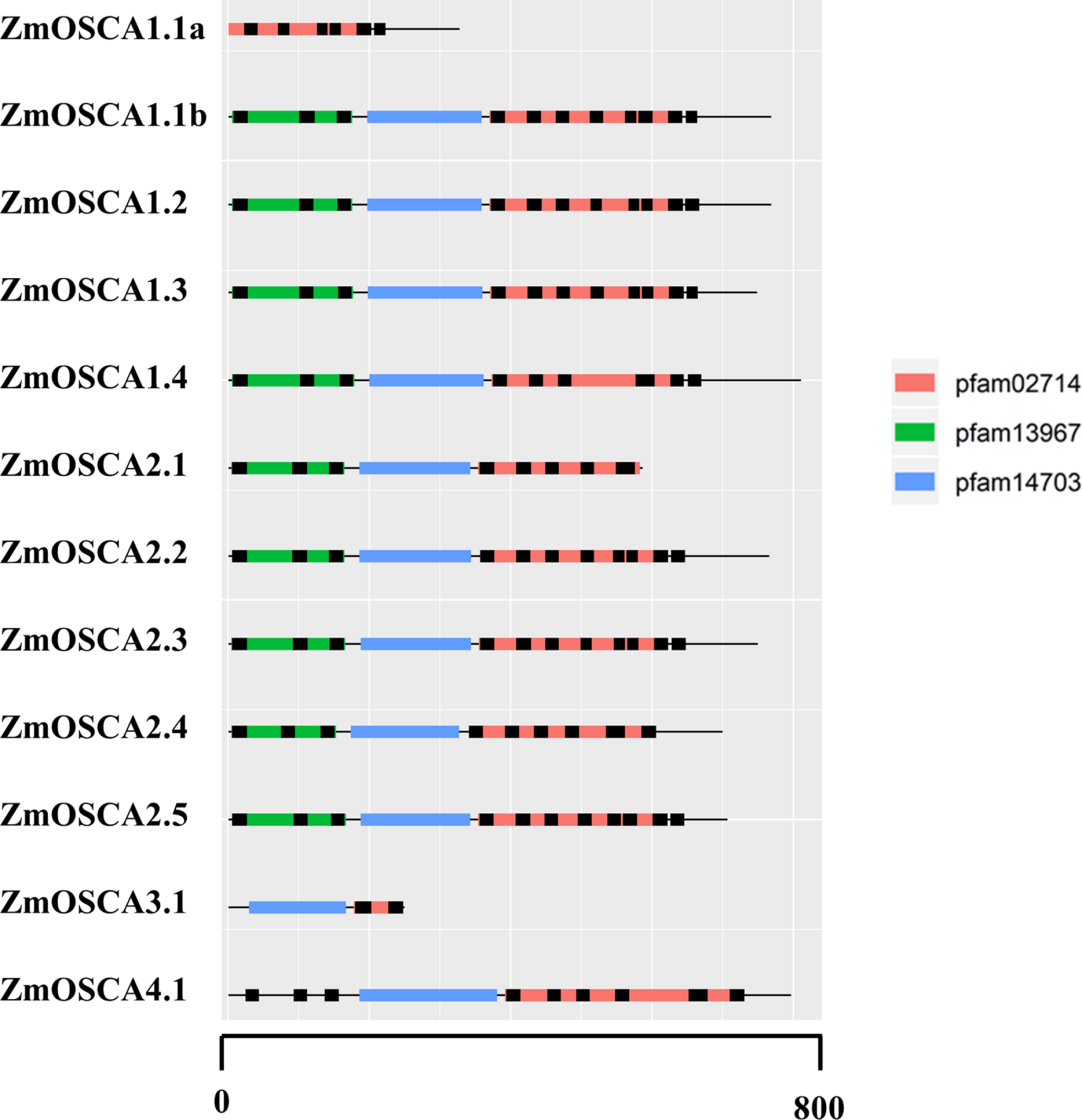
Protein domains and transmembrane regions in ZmOSCAs. Protein domains and transmembrane regions were predicted by CDD search and TMHMM. Black lines indicate the transmembrane regions. The protein domains were colored according to the color legend.

**Figure 4:**
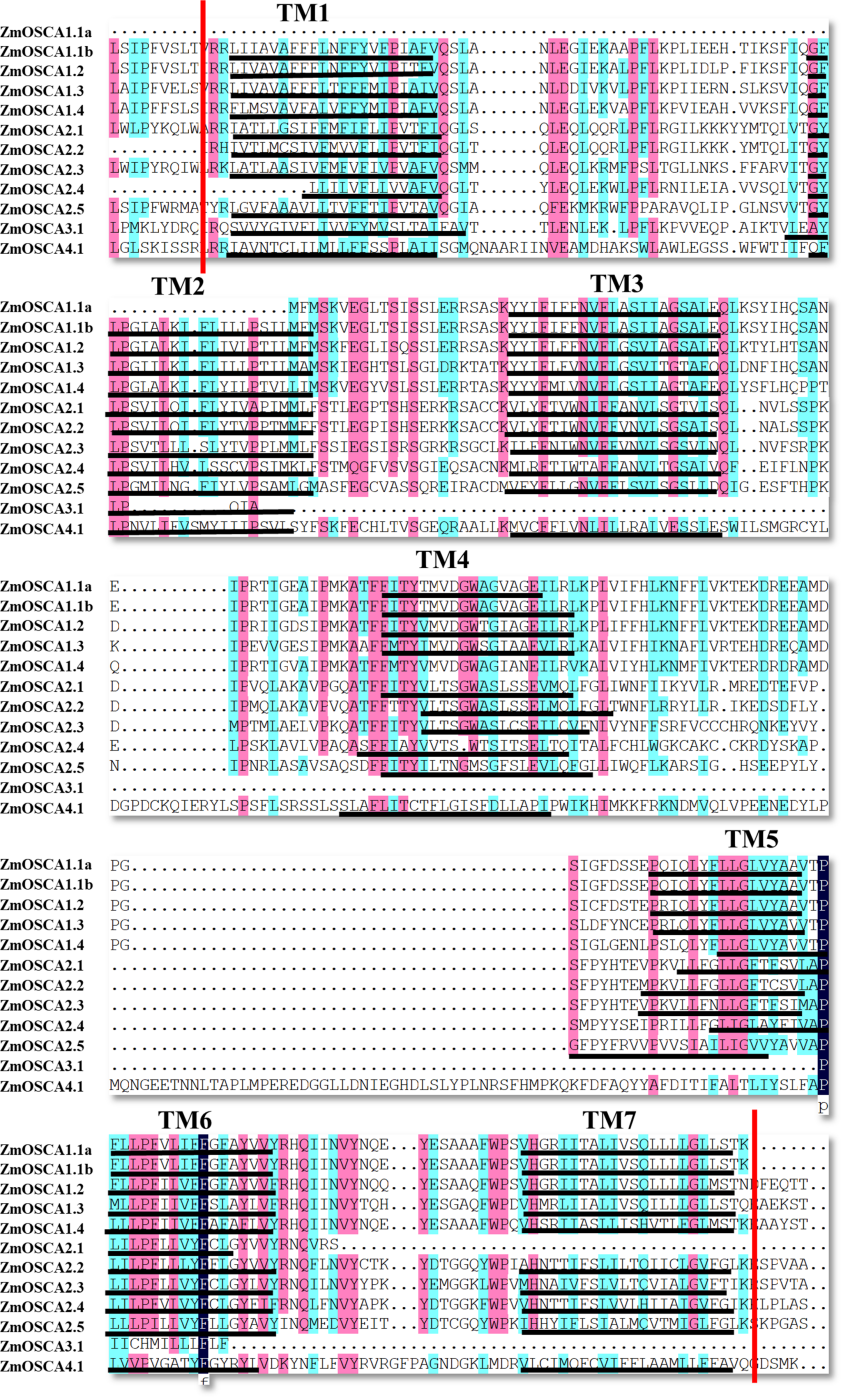
Transmembrane region of the DUF221 in ZmOSCAs. DNAman and TMHMM were used to perform multiple sequence alignments and predicte transmembrane region. TM1–TM7 are the transmembrane regions that are marked by black lines. Regions between red lines represent the conservative domain of DUF221.

Conserved Domain Database was used to identify protein domains contained in ZmOSCAs. It was found that most ZmOSCAs harbored three domains except that ZmOSCA1.1a, ZmOSCA3.1, and ZmOSCA4.1 contained two domains (Fig. 3). It is noteworthy that DUF221 is located at the C-terminal of all ZmOSCAs, indicating that this domain is not only necessary but also relatively conservative compared with other domains in the gene family. It was reported that there were 11 TMs in the OSCA protein sequences (Jojoa-Cruz et al., 2018; Liu, Wang & Sun, 2018; Maity et al., 2018; Murthy et al., 2018; Zhang et al., 2018). We found that half of the 12 ZmOSCAs had exactly 11 transmembrane regions (Fig. 3). A multiple sequence alignment was used to show the presence of TMs in DUF221 of ZmOSCAs (Fig. 4). It was found that protein domain DUF221 contained a different number of TMs, while protein domain pfam13967 contained a fixed number of TMs. However, no TMs were detected in the protein domain pfam14703 in ZmOSCAs. Different ZmOSCA members contained two to seven TMs in the DUF221 region. Most of the ZmOSCAs contained at least eight TMs with two exceptions. ZmOSCA1.1a and ZmOSCA3.1 had fewer TMs than others, suggesting that a deletion event occurred for ZmOSCA1.1a and ZmOSCA3.1.

### Expression profiles of *ZmOSCA* genes under drought stress

**Figure 5:**
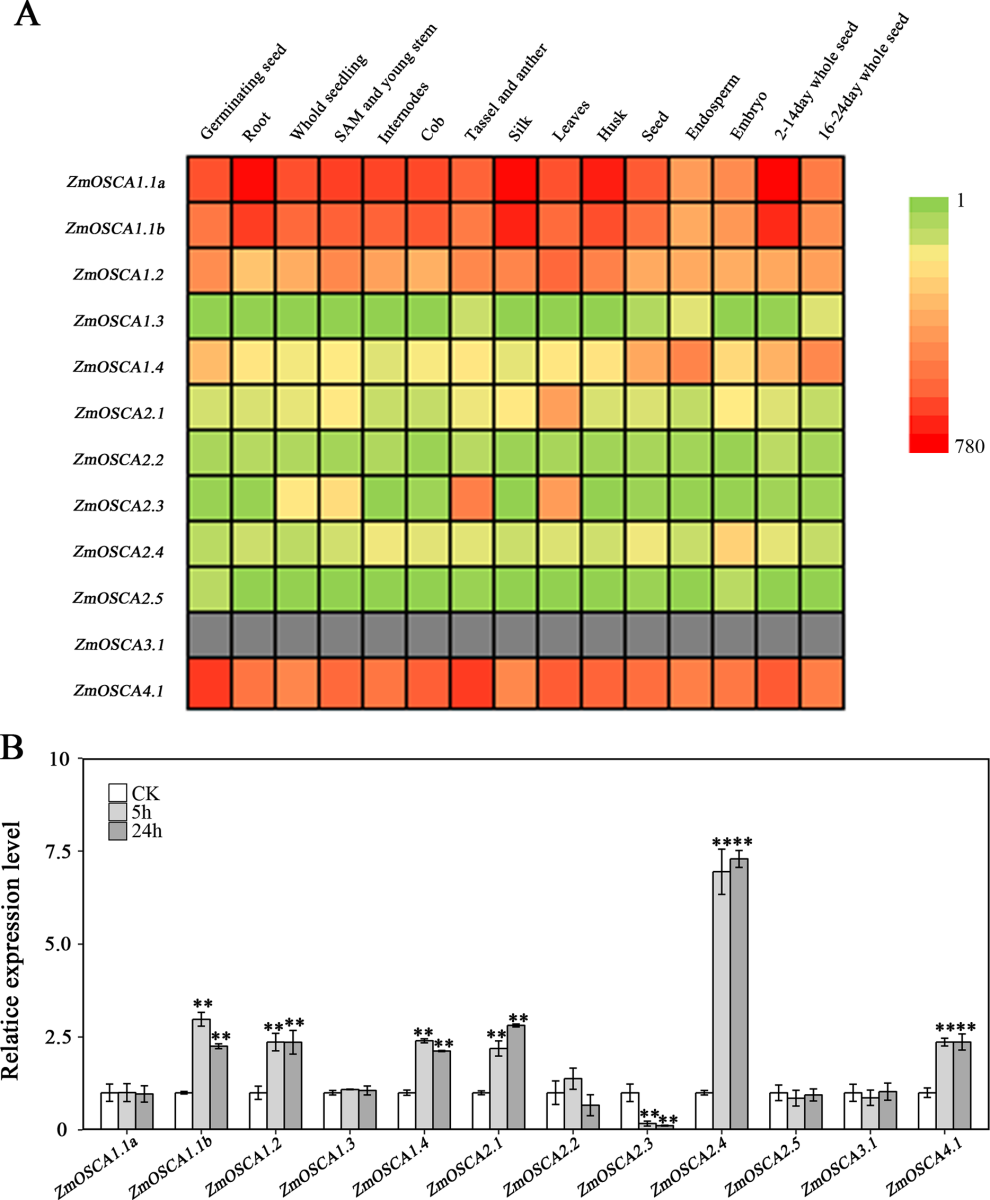
Expression profiles of *ZmOSCA*s. (A) Expression profiles of the 12 ZmOSCA genes in 15 tissues illustrated by a heat map. Different colors in the heat map represent the normalized gene expression values indicated by the scale bar. The color scale ranged from green, representing low expression, and passes through yellow and finally to red, representing high expression. The gray color shows missing data. (B) Expression profile of *ZmOSCA* genes in maize B73 seedlings under drought. Total RNA was extracted from the third leaves before and after the drought treatments. A total of 5 and 24 h, represent the collection time points, responding to relative leaf water content (RLWC) of 70% and 58%, respectively. Transcript levels of ZmUbi-2 were used as an internal control for data normalization. The represented mean and SD derived from three biological replicates. *t*-test, **P ≤ 0.01.

**Figure 6:**
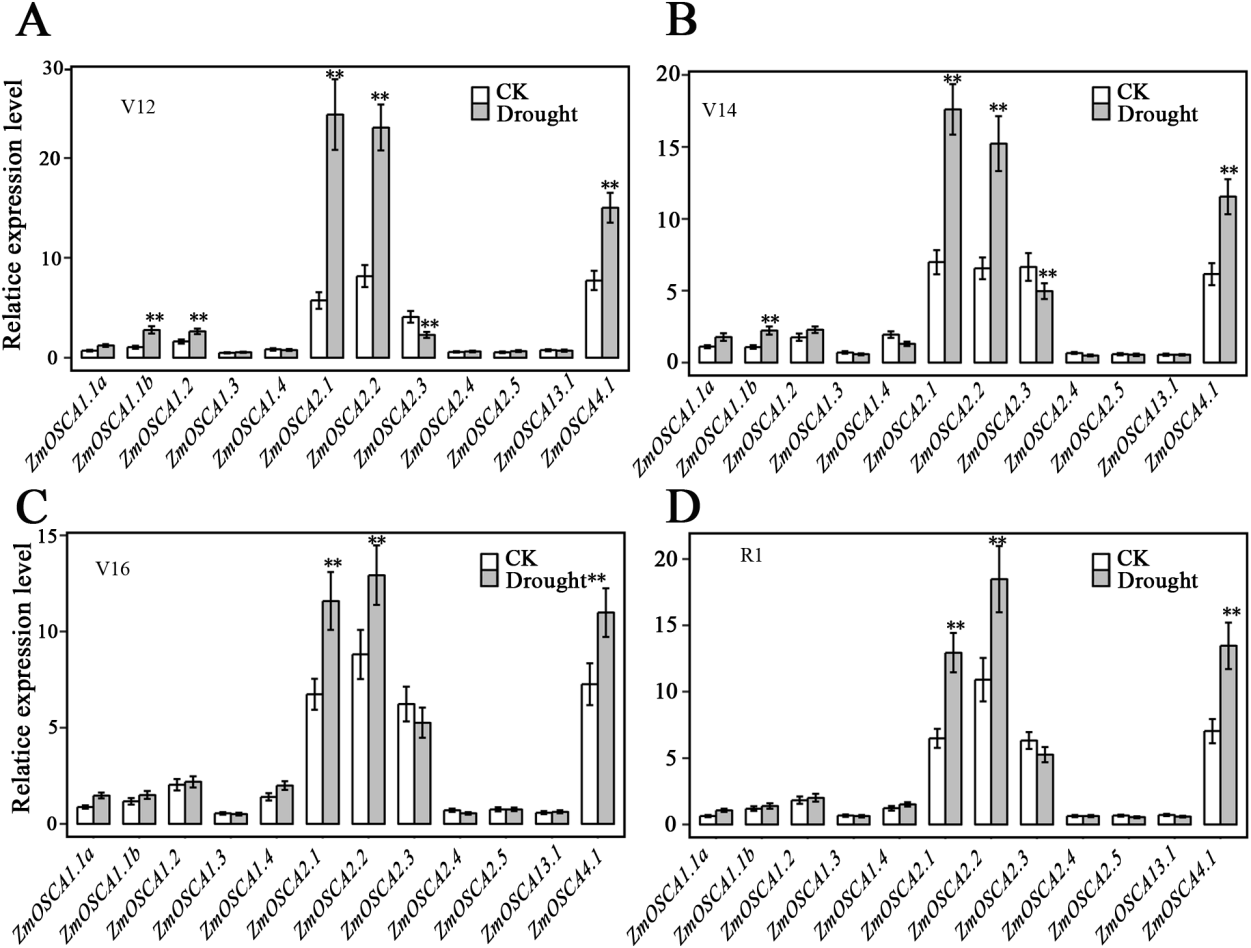
Expression patterns of *ZmOSCA* genes in maize B73 adult leaves under drought. The drought treatment started from the (eight leaves) V8 stage, while the control was well-watered. Total RNA was collected at V12 (A), V14 (B), V16 (C) and R1 (D) stage for both drought stressed and well-watered plants. Transcript levels of *ZmUbi-2* transcript levels were used served as an internal control for data normalization. Data represent the represented mean and SD derived from of three biological replicates. (*t*-test, **P ≤ 0.01).

We studied the expression profiles of the 12 ZmOSCA genes in 15 different tissues using the reported transcriptomic data (Sekhon et al., 2011). As shown in Fig. 5A, expression patterns of *ZmOSCA* genes varied greatly. Expression values of *ZmOSCA1.1a*, *ZmOSCA1.1b*, *ZmOSCA1*.2, and *ZmOSCA4*.1 were relatively higher than the rest among the tissues tested. Previous studies have shown that *OSCA*s had key roles in different aspects of plant development, especially in stress responses (Li et al., 2015). In order to obtain further insight into the roles of *ZmOSCA*s in drought tolerance, their expression profiles were monitored by quantitative real-time PCR analyses using 3-week-old leaves of maize seedlings treated with drought stress for 5 or 24 h. As shown in Fig. 5B, in the genotype of B73, more than half of expression level of the *ZmOSCA*s were regulated by drought stress, including six *ZmOSCA*s that were up-regulated significantly and one down-regulated *ZmOSCA*; the others were hardly responsive to drought stress. In addition, genes showing up- or down-regulation were found unfavorably in each of the four major clades. The results showed that proteins from different clades might have overlapping and/or antagonistic functions in the regulation of drought responses in plants. Amongst the six up-regulated *ZmOSCA* genes, in comparison to normal growth conditions, the expression of *ZmOSCA2.4* was induced more than sixfold, that being the largest folds change in relative expression levels (Fig. 5B). Five *ZmOSCA*s (*ZmOSCA1.1b*, *ZmOSCA1.2*, *ZmOSCA1.4, ZmOSCA2.1*, and *ZMOSCA4.1*) were up-regulated over twofold in response to drought (Fig. 5B). Additionally, a slightly up-regulated expression was observed in some *ZmOSCA*s following drought treatment for five hours or 24 h; these include *ZmOSCA1.1a, ZmOSCA2.2, ZmOSCA2.4, ZmOSCA2.5*, *and ZmOSCA3.1.* In contrast to the other *ZmOSCA*s, the expression level of *ZmOSCA2.3* decreased about threefold after drought treatment (Fig. 5B). Relative expression of *ZmOSCA*s were also detected in adult leaves at four growth stages, V12, V14, V16, and R1. Drought stress for the adult leaves was applied by withholding water at the V8 stage, while the corresponding control was well-watered (soil water content 40%) plants. As shown in Fig. 6, five *ZmOSCA*s were up-regulated at least one stage, and one *ZmOSCA* was down-regulated at two stages under drought. Across the four stages, the relative expression levels of *ZmOSCA2.1*, *ZmOSCA2.2*, and *ZmOSCA4.1* were up-regulated consistently. Taken together, the results clearly show the functional divergence of *ZmOSCA*s responding to drought stress in maize seedlings and adult leaves. Collectively, the data demonstrated that different *ZmOSCA*s showed variable expression patterns under drought stress, while the expression value of *ZmOSCA4.1* was highly expressed in 15 tissues and up-regulated in both seedling and adult leaf by drought stress.

### Association analysis of genetic variations in *ZmOSCA*s with drought tolerance

In order to further inquire whether the genetic variation in *ZmOSCAs* was associated with drought tolerance, a family-based association analysis was performed for these genes. At the seedling stage, the drought tolerance of the mapping population was assessed by evaluating seedling survival rate under severe drought stress. Based on previously reported methods and data (Liu et al., 2013; Wang et al., 2016), genetic polymorphism was characterized as the presence and number of SNP markers in each of the 12 *ZmOSCA*s. All the 12 identified *ZmOSCA*s were identified as polymorphic, with 14 SNPs on average for each identified gene (Table 3). As a consequence, *ZmOSCA4.1* was identified as the second most polymorphic gene, with 34 SNPs in this mapping population. Subsequently, three statistical models were used to find out significant associations between genotype and phenotype. In brief, a GLM, with the first PC_2_, and MLM incorporating both PC_2_ and a kinship matrix correcting the effect of cryptic relatedness were used in the associations. GLM method was used to conduct the single-marker analysis. PC_2_, via the first PC_2_ on SNP data, was utilized to adjust spurious associations resulted from population structure. MLM was regarded as an effective method for controlling false positives in association analysis (Yu & Buckler, 2006; Yu et al., 2006). Subsequently, candidate gene association analysis identified significant associations between genetic variation of *ZmOSCA4.1* with drought tolerance under different models with a *P*-value ≤ 0.01 (Table 3). The results appeared to show that *ZmOSCA4.1* might be an important candidate gene for maize drought tolerance.

**Table 3:** Number of significantly associated genetic variation in *ZmOSCA* genes with drought tolerance at the seedling stage.

Gene ID	Gene name	Polymorphic number*	GLM	PC_2_	PC_2_ + K
			*P* ≤ 0.01	*P* ≤ 0.01	*P* ≤ 0.01
GRMZM2G064189	*ZmOSCA1.1a*	4	0	0	0
GRMZM2G021194	*ZmOSCA1.1b*	22	0	0	0
GRMZM2G456000	*ZmOSCA1.2*	11	4	0	0
GRMZM2G181206	*ZmOSCA1.3*	14	0	0	0
GRMZM2G128641	*ZmOSCA1.4*	42	7	0	0
GRMZM2G163059	*ZmOSCA2.1*	12	2	0	0
GRMZM2G409093	*ZmOSCA2.2*	3	0	0	0
GRMZM2G164470	*ZmOSCA2.3*	2	0	0	0
GRMZM2G039186	*ZmOSCA2.4*	13	0	0	0
GRMZM2G402708	*ZmOSCA2.5*	4	0	0	0
GRMZM2G162253	*ZmOSCA3.1*	7	0	0	0
GRMZM2G059891	*ZmOSCA4.1*	34	10	2	3

**Note:**

* MAF (minor allele frequency) ≥0.05.

## Discussion

Previous study has indicated that *OSCA1*, containing the domain DUF221, is an osmosensor in *Arabidopsis* (Yuan et al., 2014). Subsequently, studies have been done to identify *OSCA* family members in *Arabidopsis* and rice, which lead to the identification of 15 *AtOSCA*s and 11 *OsOSCA*s, respectively (Yuan et al., 2014; Li et al., 2015). However, no detailed analysis has been performed for the *OSCA* gene family in maize, especially its expression profile under drought stress. Moreover, it is not yet known which *ZmOSCA* is directly associated with the diversity of drought tolerance in maize. Therefore, we carried out this study to address these questions. Our findings will provide fundamental factors that may be used to facilitate the genetic enhancement of drought tolerance in maize, as well as increase our understanding of the role of this gene family under drought.

In our study, we identified 12 *ZmOSCA*s in maize, and then we systematically analyzed their phylogenetic and synteny relationship with rice, maize, and sorghum genomes (Figs. 1 and 2). After MRCA analysis, we found that the *Arabidopsis* genome possessed the largest number of *OSCA*s, whilst the maize genome possessed three gene losses compared to *Arabidopsis* and one gene gain compared to the rice genome. The number of *OSCA*s within the maize, sorghum, and rice genomes did not vary much, indicating that the majority of *OSCA*s in maize, sorghum, and rice genomes undergo relatively conserved evolutionary history after their divergence. As shown in Fig. 1, 12 maize genes were clustered into four clades as reported previously (Yuan et al., 2014). Gene numbers in clades 2, 3, and 4 were identical between maize, sorghum, and rice genomes. As shown in Fig. 1, the maize genome possessed one more *OSCA*, namely *ZmOSCA1.1a*, having the fewest number of exons in clade 1. But, in the synteny analysis, we did not find the synteny blocks related to *ZmOSCA1.1a*, suggesting that *ZmOSCA1.1a* may derive from duplication after the divergence of the maize, sorghum, and rice genomes and possess functional differentiation from other *ZmOSCA*s. Meanwhile, we found that, despite *ZmOSCA3.1*, *OsOSCA3.1*, and *Sobic.002G034500* were clustered together (Fig. 1), *ZmOSCA3.1* was absent from the synteny analysis (Fig. 2). Interestingly, *ZmOSCA3.1* was constituted by two exons. The results suggest that *ZmOSCA3.1* may exit before the maize, sorghum, and rice genomes split, but evolved in a different direction from *OsOSCA3.1* and *Sobic.002G034500*. Previous studies showed that there were 11 TMs in the OSCA protein sequences (Jojoa-Cruz et al., 2018; Liu, Wang & Sun, 2018; Maity et al., 2018; Murthy et al., 2018; Zhang et al., 2018), and each OSCA protein in the Arabidopsis genome contained 11 TMs (Jojoa-Cruz et al., 2018). However, only half of the 12 ZmOSCAs had exactly 11 transmembrane regions, demonstrating that ZmOSCAs might possess a greater genetic variation during the evolution. Protein domain and transmembrane regions analysis also revealed that *ZmOSCA1.1a* and *ZmOSCA3.1* contained fewer domains and TMs compared with others (Figs. 3 and 4). Obviously, deletions in *ZmOSCA1.1a* and *ZmOSCA3.1* occurred independently during maize evolution. However, both *ZmOSCA1.1a* and *ZmOSCA3.1* did not respond to drought treatment, suggesting that they may not have a function in drought resistance.

To our knowledge, although the relationship between OSCA proteins and stresses has been reported (Zhao et al., 2015), the dynamic drought-responsive expression patterns of *ZmOSCA*s were still obscure. Expression pattern analysis of *ZmOSCA*s helped us to understand their possible functions and offer a thorough foundation for future functional studies. Generally, *ZmOSCA*s exhibited differential expression under drought stress in maize seedlings and adult leaves, not only amongst sub-groups but also amongst members within the same sub-groups, suggesting that these *ZmOSCA*s may have diverse functions. Our results showed that the relative expression levels of six *ZmOSCA*s were significantly up-regulated and expression of one *ZmOSCA* was clearly down-regulated (Fig. 5), indicating that these genes might serve as key mediators of drought stress responses. In other research, the relative expression of *OsOSCA1.1*, *OsOSCA1.2*, *OsOSCA2.1*, *OsOSCA2.4*, *OsOSCA2.5*, and *OsOSCA4.1* were up-regulated by PEG treatment (Li et al., 2015). Interestingly, in this study, we found that six *ZmOSCA*s, including *ZmOSCA1.1b*, *ZmOSCA1.2*, *ZmOSCA1.4*, *ZmOSCA2.1*, *ZmOSCA2.4,* and *ZmOSCA4.1,* could be up-regulated by drought stress (Fig. 5). When we analyzed the expression pattern of *ZmOSCA*s in adult leaves at four stages, *ZmOSCA2.1*, *ZmOSCA2.2*, and *ZmOSCA4.1* were found to be up-regulated consistently (Fig. 6) at both seedlings and adult leaf. Notably, the relative expression level of the rice orthologs of ZmOSCA2.1, ZmOSCA2.2, and ZmOSCA4.1 were also up-regulated by drought stress (Li et al., 2015), demonstrating that these genes may share a conserved function.

To date, no study is known which detects the association between genetic variations in *ZmOSCA*s and drought tolerance. To answer this question, the genetic polymorphism amongst all of the 12 *ZmOSCA*s were analyzed. Under the three models, *ZmOSCA4.1* was consistently the most significantly associated with drought tolerance (*P*-value ≤ 0.01, Table 3). Furthermore, in response to drought stress, the expression of *ZmOSCA4.1* was induced, indicating that this gene might be engaged in resistance to drought stress. Further detailed analysis of biochemical and molecular functions of *ZmOSCA4.1* will contribute to our understanding of *OSCA* function.

## Conclusions

In this report, 12 *ZmOSCA* genes were uncovered in the maize. Synteny and phylogenetic relationship analyses were performed, and it was found that the *ZmOSCA* gene family shared a conserved evolutionary history. Protein domain and transmembrane analysis indicated that most of the 12 ZmOSCAs shared similar structures with their homologs. Differential expression analysis under drought at various stages, as well as the expression profiles in 15 tissues, revealed a functional divergence of *ZmOSCA* genes. Notably, the expression level of *ZmOSCA4.1* being up-regulated in both seedlings and adult leaves. Importantly, significant associations between genetic variation in *ZmOSCA4.1* and drought tolerance were found at the seedling stage. Our research will enhance understanding of the role of *ZmOSCAs* under drought.

## Supplemental Information

10.7717/peerj.6765/supp-1 Supplemental Information 1 Gene and protein sequences used to construct phylogenetic tree. Raw data for gene and protein sequences used to construct phylogenetic tree in Fig. 1.

10.7717/peerj.6765/supp-2 Supplemental Information 2 Collinear segments between maize, rice, and sorghum genomes. Raw data for collinear segments between maize, rice, and sorghum genomes in Fig. 3.

## Additional Information and Declarations

### Data Availability

The following information was supplied regarding data availability: The raw measurements are available in Supplemental Files 1 and 2.
